# Molecular Landscape of Classical Hodgkin Lymphoma: From Genetic Lesions to Biologically Defined Subtypes

**DOI:** 10.3390/cancers18203256

**Published:** 2026-10-09

**Authors:** Maria Cristina Pirosa, Zhihua Yao, Davide Rossi

**Affiliations:** 1 Laboratory of Experimental Hematology, Institute of Oncology Research, 6500 Bellinzona, Switzerland; 2 Clinic of Hematology, Oncology Institute of Southern Switzerland, Ente Ospedaliero Cantonale, 6500 Bellinzona, Switzerland; 3 Faculty of Biomedical Sciences, Università della Svizzera Italiana, 6900 Lugano, Switzerland; 4 Department of Internal Medicine, Affiliated Cancer Hospital of Zhengzhou University & Henan Cancer Hospital, Zhengzhou 450008, China

**Keywords:** classical Hodgkin lymphoma, Hodgkin and Reed–Sternberg cells, genomic landscape, molecular classification, circulating tumor DNA, tumor microenvironment, Epstein–Barr virus, immune evasion

## Abstract

Hodgkin lymphoma (HL) represents a unique lymphoid malignancy characterized by the presence of rare malignant Hodgkin and Reed–Sternberg (HRS) cells embedded within a highly complex and immunologically active tumor microenvironment. Over the past decade, advances in genomic and transcriptomic technologies have substantially refined the understanding of HL pathogenesis. Recurrent genetic alterations affecting key oncogenic pathways, including NF-κB, JAK/STAT, and PI3K/AKT signaling, cooperate with widespread mechanisms of immune evasion to sustain tumor cell survival and proliferation. Epstein–Barr virus infection contributes to lymphomagenesis in a significant subset of cases by providing surrogate survival signals, modulating immune interactions and targeting the telomere-shelterin complex through LMP1. In parallel, emerging evidence highlights the central role of the tumor microenvironment, which actively supports malignant cell growth through direct cellular interactions, cytokine signaling, and metabolic and immune-regulatory mechanisms. Recent studies using circulating tumor DNA (ctDNA) profiling and single-cell approaches have enabled non-invasive genomic characterization and revealed biologically distinct molecular subgroups, further underscoring the interplay between tumor genetics and immune contexture. These insights are reshaping disease classification and opening new avenues for biomarker-driven therapeutic strategies. A deeper integration of molecular, immunological, and microenvironmental features will be crucial to optimize risk stratification and to develop more precise and less toxic treatments for patients with HL.

## Simple Summary

Classical Hodgkin lymphoma is a highly curable cancer of the lymphatic system, but its biology is unusually complex because only a small number of cancer cells are surrounded by many normal immune cells. Recent advances in genetic and molecular technologies have greatly improved our understanding of how this disease develops and interacts with the immune system. This review summarizes current knowledge on the genetic changes that drive the disease, the role of Epstein–Barr virus in some patients, and the contribution of the tumor microenvironment to cancer growth and immune escape. It also highlights how new approaches, such as circulating tumor DNA analysis and single-cell technologies, are improving disease classification and identifying new biomarkers. A better understanding of these biological mechanisms may support the development of more effective and less toxic therapeutic strategies and guide future research in Hodgkin lymphoma.

## 1. Introduction

Hodgkin lymphoma (HL) is one of the most frequent lymphomas in the Western world, with an annual incidence of approximately three new cases per 100,000 individuals. It displays a characteristic bimodal age distribution, with a first peak in young adults between 20 and 30 years of age and a second peak between 50 and 70 years [1]. Epstein–Barr virus (EBV) infection is implicated in approximately 25–40% of Hodgkin lymphoma cases [2], with a higher prevalence observed in older patients. Additional risk factors include autoimmune disorders and immunodeficiency states, such as HIV infection [1,3].

HL is subdivided into two biologically distinct entities: classical Hodgkin lymphoma (cHL) and nodular lymphocyte-predominant Hodgkin lymphoma (NLPHL) [4]. cHL is characterized by Hodgkin/Reed–Sternberg (HRS) cells, large atypical cells of B-cell origin. Immunophenotypically, HRS cells consistently express CD30, frequently express CD15 (75–80%), and show weak expression of PAX5 in approximately 95% of cases, whereas CD20 expression is detected in only a minority of cases (up to 20%) [5,6]. Both the fifth edition of the World Health Organization Classification of Hematolymphoid Tumors (WHO-HAEM5) [5] and the International Consensus Classification (ICC) [6] retain the four histological subtypes of cHL established in the previous WHO classification: nodular sclerosis (NSCHL), mixed cellularity (MCCHL), lymphocyte-rich (LRCHL), and lymphocyte-depleted (LDCHL). Although this histological subclassification does not currently translate into distinct therapeutic strategies, the individual subtypes are associated with specific clinical and biological features, including differences in patient age, EBVstatus, and patterns of nodal involvement.

NLPHL, in contrast, represents a distinct clinicopathological entity with morphological, immunophenotypic, and biological features that differ substantially from those of cHL [3]. Rather than HRS cells, NLPHL is characterized by large neoplastic lymphocyte-predominant (LP) cells which have folded or multilobulated nuclei, retain a functional B-cell program, typically express B-cell markers such as CD20, and usually lack CD30 expression. While WHO-HAEM5 continues to classify NLPHL within the Hodgkin lymphoma family, the ICC recognizes it as “nodular lymphocyte-predominant B-cell lymphoma,” reflecting its preserved B-cell phenotype and its biological relationship to T-cell/histiocyte-rich large B-cell lymphoma [5,6].

Clinically, most patients with cHL present with painless superficial lymphadenopathy, most commonly involving the cervical and supraclavicular regions, with disease predominantly affecting supradiaphragmatic sites. Mediastinal involvement is observed in approximately 60% of patients, with bulky disease occurring in 20–25% of cases and potentially causing symptoms such as cough, dyspnea, or, less commonly, superior vena cava syndrome. Isolated infradiaphragmatic involvement is uncommon, occurring in fewer than 10% of patients. Approximately 30–40% of patients present with systemic B symptoms, including unexplained fever (>38.3 °C), drenching night sweats, and unintentional weight loss exceeding 10% of body weight within the preceding 6 months [7].

Over the past decades, treatment strategies for cHL have evolved substantially, driven by the implementation of PET-adapted approaches and the introduction of novel agents, including the anti-CD30 antibody–drug conjugate brentuximab vedotin (BV) and the PD-1 checkpoint inhibitors nivolumab and pembrolizumab. These agents have been progressively incorporated into therapeutic regimens across different disease settings, including unfavorable early-stage, advanced-stage, and relapsed or refractory cHL, contributing to substantial improvements in patient outcomes. Given that cure rates now approach 90%, current treatment strategies increasingly focus on de-escalation approaches aimed at maintaining high efficacy while minimizing treatment-related toxicity and long-term morbidity, particularly in the predominantly young population affected by cHL [7,8,9].

A hallmark of cHL is its unusual tissue composition. The malignant Hodgkin and Reed–Sternberg (HRS) cells, represent 1–5% of the total cellular infiltrate within affected lymph nodes in most cases.

The tumor mass is instead dominated by an extensive and heterogeneous inflammatory microenvironment composed of T cells, B cells, macrophages, eosinophils, and stromal elements [10,11]. The rarity of tumor cells within affected lymph nodes has long hindered their molecular analysis, which is one reason why the biology and pathogenesis of cHL remained enigmatic for decades.

In the late 1990s, the introduction of laser capture microdissection enabled the first molecular analyses of HRS cells [12,13]; however this method is labor-intensive and may compromise DNA integrity. Subsequent methodological advances, including flow cytometric purification of HRS cells from fresh tissue and FACS-based enrichment from FFPE samples [14], enabled higher-quality genomic profiling and paved the way for genome-wide approaches. Three landmark whole-exome sequencing studies of purified HRS cells [15,16,17] defined the broader mutational landscape of classical HL. More recently, whole-genome sequencing studies [18,19] have further expanded this framework by uncovering structural variants and non-coding alterations. In parallel, analysis of circulating tumor DNA (ctDNA), released by HRS cells into the plasma despite their scarcity within the tumor tissue, has emerged as a minimally invasive alternative that overcomes many of these technical limitations. Beyond accurately recapitulating the genomic profile of HRS cells, ctDNA enables serial sampling, longitudinal disease monitoring, and assessment of minimal residual disease [20,21,22,23,24].

In this review, we discuss the major advances in the understanding of Hodgkin lymphoma biology and its genetic landscape.

## 2. Cellular Origin of HRS Cells

Comprehensive molecular analyses of isolated HRS cells have consistently identified clonally rearranged and somatically mutated immunoglobulin genes, demonstrating that cHL arises from germinal center (GC) B cells. Because somatic hypermutation occurs exclusively in antigen-stimulated B cells within the GC, these findings establish a GC experience of the HRS precursors [25,26,27,28]. Remarkably, in approximately one quarter of cases, the immunoglobulin genes harbor destructive or “crippling” mutations, such as nonsense mutations or frameshifts, that abolish functional B-cell receptor (BCR) expression. Under physiological GC conditions, such mutations would result in apoptosis, as B cells with impaired antigen affinity or unstable immunoglobulin structure are negatively selected. These observations support a model in which HRS cells derive from pre-apoptotic GC B cells that would normally be eliminated but instead acquire additional transforming events enabling survival despite loss of BCR signaling [29,30].

In EBV-positive cases, viral proteins such as LMP1 and LMP2A may provide surrogate CD40- and BCR-like signals, respectively, thereby facilitating the rescue of BCR-deficient GC B cells from apoptosis [31]. Notably, early experimental evidence also demonstrated a direct role for LMP1 in promoting the characteristic Reed–Sternberg cell phenotype. Expression of the LMP1 oncoprotein in the EBV-negative Hodgkin lymphoma cell line L-428 was strongly associated with the development of multinucleated RS-cell morphology, providing morphological evidence that LMP1 can contribute to RS-cell formation [32]. Thus, in a subset of cHL cases, EBV may contribute not only to the survival of otherwise apoptosis-prone B-cell precursors but also to the acquisition of characteristic features of the HRS phenotype.

At the same time, HRS cells undergo extensive transcriptional reprogramming, resulting in the near-complete silencing of the B-cell gene expression program. Although the mechanisms underlying suppression of the B-cell transcriptional program in HRS cells are not yet fully elucidated, several contributing processes have been identified. These include reduced expression of key B-cell–specific transcription factors (such as OCT2, PU.1, and BOB1), aberrant activation of transcriptional repressors that inhibit B-cell gene expression (including ID2, ABF1, NOTCH1, and STAT5), and epigenetic silencing of B-cell–associated genes [33].

Rare cases with apparent T-cell derivation have been reported [34,35], but these are exceptional, and it remains unclear whether they represent true cHL or rather a form of T-cell lymphoma. Recent molecular studies have helped clarify this issue. In particular, integrated analysis of immunoglobulin and T-cell receptor gene rearrangements together with lymphoma-associated mutations has shown that some cases initially classified as cHL, particularly those harboring clonal T-cell receptor rearrangements, actually represent T-cell lymphomas with HRS-like cells mimicking cHL [36].

## 3. Genetic Lesions in HRS Cells

**Figure 1:**
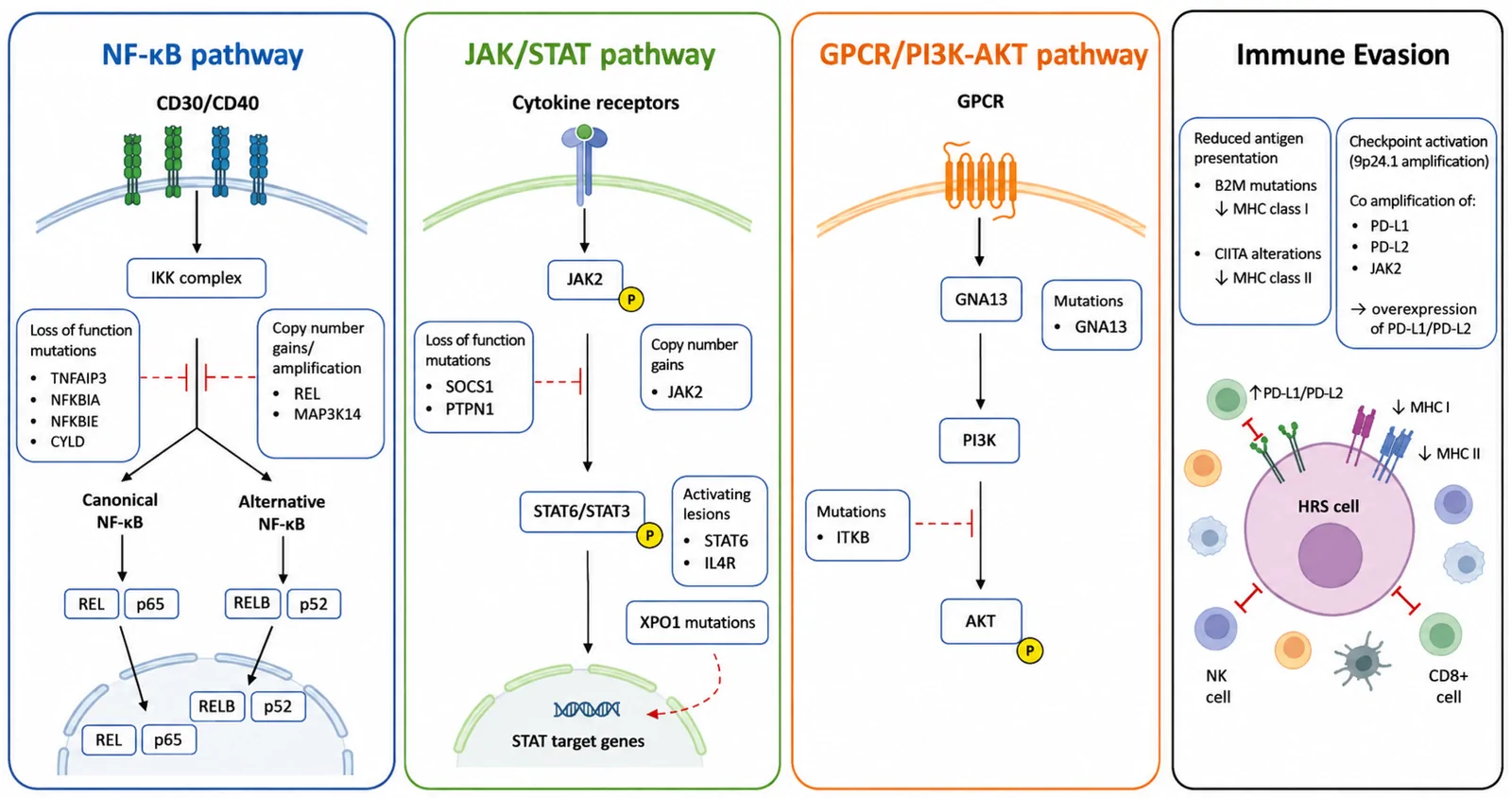
Major genetic alterations and signaling pathways in classical Hodgkin lymphoma. Schematic representation of the major signaling pathways recurrently affected by genetic lesions in Hodgkin and Reed–Sternberg (HRS) cells, including the canonical and alternative NF-κB pathways, the JAK/STAT pathway, and the G protein-coupled receptor (GPCR)/PI3K–AKT pathway. Major genetic mechanisms of immune evasion are also depicted, including alterations leading to reduced MHC class I and II antigen presentation and 9p24.1 copy-number alterations resulting in increased PD-L1/PD-L2 expression. Representative genes affected by loss-of-function mutations, activating mutations, or copy-number gains/amplifications are indicated within each pathway. Figure partially created in BioRender. Pirosa, M. C. (2026) https://BioRender.com/rsdsipf (accessed on 1 August 2026).

The genetic pathogenesis of cHL is characterized by recurrent chromosomal alterations and somatic mutations affecting key signaling networks in HRS cells [37,38,39]. Constitutive activation of the NF-κB pathway, involved in up to 40% of cHL cases, represents a central oncogenic mechanism and is observed in the majority of cases, resulting from inactivating mutations in negative regulators such as *NFKBIA*, *NFKBIE*, *TNFAIP3*, and *CYLD*, as well as from genomic gains or amplifications involving positive pathway components including *REL* and *MAP3K14* [40]. These lesions affect both canonical and alternative NF-κB signaling cascades and converge to sustain transcriptional programs that promote tumor cell survival, proliferation, cytokine production, and resistance to apoptosis, thereby facilitating the persistence of genetically crippled germinal center B cells that would otherwise undergo programmed cell death (Figure 1).

A second major hallmark of cHL biology is deregulated JAK/STAT signaling, driven by recurrent mutations affecting pathway inhibitors such as *SOCS1* [41] and *PTPN1* [42], together with copy number gains involving *JAK2* [38,43], detected in approximately 40% of cases, and activating mutations or gains affecting *STAT6*. These alterations result in constitutive STAT activation, reinforcing proliferative and anti-apoptotic signaling while contributing to immune modulation through enhanced cytokine responsiveness and microenvironmental crosstalk [38]. In addition, activating *XPO1* mutations, occurring in about 20–25% of cHL cases [16,44], promote nuclear accumulation of STAT proteins and have been associated with a trend toward inferior progression-free survival, particularly in patients harboring the E571K hotspot mutation [44] (Figure 1).

Notably, amplification of the chromosome 9p24.1 locus frequently co-targets PD-L1, PD-L2, and JAK2, thereby functionally linking oncogenic signaling with immune escape [38].

Additional oncogenic inputs arise from alterations in PI3K/AKT and G-protein–coupled receptor (GPCR) signaling pathways, including recurrent mutations in *GNA13* and genetic lesions affecting *ITPKB*, which collectively enhance downstream AKT activation, metabolic fitness, and survival capacity of HRS cells [10] (Figure 1).

Genetic alterations enabling immune evasion constitute a further fundamental pathogenic axis in cHL and include reduced or absent expression of MHC class I molecules, often resulting from β2-microglobulin (*B2M*) mutations [15,45], as well as alterations affecting the MHC class II transcriptional machinery, such as *CIITA* rearrangements [46]. These mechanisms are frequently accompanied by structural or copy number alterations leading to overexpression of PD-L1/PD-L2, ultimately impairing recognition and elimination of tumor cells by cytotoxic T lymphocytes and natural killer cells [47] (Figure 1). Collectively, this spectrum of immune-modulatory lesions provides a biological rationale for the remarkable clinical sensitivity of cHL to PD-1 blockade, highlighting immune evasion as a central driver of lymphoma pathobiology.

Additional recurrent genetic lesions further contribute to the molecular heterogeneity of classical Hodgkin lymphoma. In particular, *TP53* mutations, detected in approximately 5–10% of patients, are associated with increased genomic instability and higher overall mutational burden, underscoring the critical role of p53 in maintaining genomic integrity [48,49].

## 4. Genetic Clusters in HL

The advent of ctDNA sequencing has enabled genomic profiling of larger cohorts of cHL despite the scarcity of tumor cells in tissue biopsies [22,24]. More recently, this approach has substantially reshaped our understanding of the genomic architecture of cHL, allowing the identification of distinct biologically relevant genetic clusters.

Leveraging plasma-based genotyping, Alig and colleagues applied an unsupervised approach integrating somatic mutations and copy-number alterations to 293 patients with adequate ctDNA levels, identifying two molecular subtypes of cHL [20]. The H1 subtype (68%) was characterized by a high single-nucleotide variant mutational burden and recurrent alterations affecting canonical NF-κB (*TNFAIP3*, *B2M*), JAK/STAT (*SOCS1*, *STAT6*, *CSF2RB*), and PI3K signaling pathways, together with transcriptional signatures consistent with cytokine-driven tumor–microenvironment interactions. In contrast, the H2 subtype (32%) displayed a markedly higher burden of somatic copy-number alterations, recurrent *TP53* and *KMT2D* mutations, and widespread chromosomal instability despite a lower mutational burden. Clinically, H2 tumors were enriched for EBV-positive and mixed-cellularity cases, occurred more frequently in older and male patients, exhibited significantly higher baseline ctDNA levels, and were associated with inferior progression-free survival, with the adverse prognostic effect remaining significant after adjustment for ctDNA levels. External validation in an independent cohort confirmed the robustness and mutual exclusivity of the H1 and H2 genomic signatures. Biologically, H1 tumors appeared to be driven predominantly by cytokine signaling and immune escape through frequent MHC class I loss, whereas H2 tumors exhibited features of genomic instability, including recurrent 9p24.1 amplifications encompassing CD274 (PD-L1), thereby promoting PD-1 axis activation and T-cell exhaustion as an alternative mechanism of immune escape [20].

Complementing these findings, a prospective ctDNA sequencing analysis conducted within large prospective clinical trial cohorts proposed a biologic classification of Hodgkin lymphoma into three distinct subtypes: inflammatory immune-escape HL (44.7%), virally driven HL (23.0%), and oncogene-driven HL (32.3%). These entities differed not only in their genomic landscapes but also in their tumor microenvironment composition and clinical behavior [21].

The inflammatory immune-escape subtype was characterized by frequent copy number alterations, particularly high-level 9p24.1 amplifications involving the PD-L1/PD-L2 locus, together with an immune-rich microenvironment enriched in B cells, follicular helper T cells, and regulatory T cells.

In contrast, the virally driven subtype exhibited a low tumor mutational burden (TMB), frequent EBV and/or HHV-6 positivity, and a myeloid- and cytotoxic cell-rich microenvironment, including macrophages, dendritic cells, NK cells, eosinophils, and CD8^+^ T cells, suggesting an antiviral immune response. Finally, the oncogene-driven subtype exhibited the highest TMB and recurrent mutations affecting oncogenic pathways, including *TNFAIP3*, *ITPKB*, *SOCS1*, and *B2M*, together with a relatively immune-cold microenvironment. Patients harboring the oncogene-driven subtype experienced the most favorable progression-free survival, whereas the inflammatory immune-escape subtype was associated with the highest risk of relapse [21]. Interestingly, in the context of the classification proposed by Alig et al., the oncogene-driven subtype largely corresponds to H1, whereas H2 may comprise both inflammatory immune-escape and virally driven HL [21].

More recently, a comprehensive ctDNA-based genomic study further expanded this framework by showing that the molecular classification of cHL is primarily driven by distinct mechanisms of genomic instability rather than by the clustering of recurrently mutated pathways [23]. Unsupervised analysis identified two main clusters and a small residual subgroup, named C0 (2%), comprising cases lacking class-defining genomic alterations. Cluster C1 (63%) was a hypermutated subtype characterized by a high mutational burden, activation-induced cytidine deaminase (AID)-associated mutational signatures, microsatellite instability (MSI)-related features, increased predicted neoantigen load, and frequent whole-genome duplication (WGD); while C2 (35%), was defined by predominant chromosomal instability with an extensive burden of somatic copy-number alterations and a lower mutation density. Notably, both C1 and C2 shared the hallmark genomic lesions of cHL, including recurrent STAT6 mutations and gains at 2p/2p16.1 and 9p/9p24.2, while displaying similar baseline clinical characteristics, including age, sex, histologic subtype, EBV status, B symptoms, clinical stage, and ctDNA levels. Beyond coding alterations, the study also demonstrated that noncoding regulatory mutations were highly prevalent (83% of cases), identifying recurrent mutations within the *BCL6* intronic super-enhancer (approximately 31% of patients) as a novel mechanism of BCL6 deregulation [23].

Table 1 summarizes and compares the main characteristics of the three studies on molecular clusters in Hodgkin lymphoma.

Unlike previous ctDNA-based classifications, Aoki and colleagues developed a novel molecular classification of cHL, termed HLGen, based on an integrated analysis of purified HRS cells combining genomic profiling, spatial transcriptomics, and imaging mass cytometry to link tumor genetics with the immune microenvironment [50]. In a discovery cohort of 114 fresh-frozen tumor samples from patients treated with ABVD or ABVD-like regimens, the authors first confirmed the recurrent genomic landscape of cHL, including frequent mutations in *SOCS1*, *STAT6*, *TNFAIP3*, and *B2M*, together with recurrent copy number alterations involving 9p24.1 (*JAK2*, *CD274*, *PDCD1LG2*), 2p15 (*REL*, *BCL11A*), and 6q23.3 (*TNFAIP3*). Distinct genomic profiles were associated with specific clinical and biological features: EBV-negative tumors were enriched for *BCL7A* and *TNFAIP3* mutations and 2p15 gains, thymic cHL preferentially harbored *STAT6*, *GNA13*, *ITPKB*, and *TBL1XR1* mutations, while *STAT6* alterations were associated with inferior progression-free survival in younger patients. Building upon these observations, the HLGen classification identified four biologically distinct subtypes (CST, CN913, STB, and CN2P). These subgroups are defined by characteristic genomic alterations, including *CSF2RB/TNFAIP3* mutations (CST), 9p24.1 amplification with 13q deletion (CN913), *STAT6/B2M* mutations (STB), and 2p15 gains (CN2P) and are further distinguished by unique transcriptional programs, immune microenvironment composition, and clinical features. Mechanistically, the CST subtype is driven by truncating *CSF2RB* mutations leading to aberrant *STAT5* signaling and is characterized by a microenvironment enriched in FOXP3^+^ regulatory T cells and CCR4^+^ CD4^+^ T cells. CN913 is enriched for EBV-positive disease, activation of interferon and NF-κB signaling, and a microenvironment rich in LAG3^+^ CD4^+^ T cells and PD-L1^+^ macrophages. STB exhibits *STAT6/B2M* alterations, activation of TGF-β signaling, and an immune-cold phenotype characterized by a relative paucity of granzyme B-positive cytotoxic CD8^+^ T cells. Finally, CN2P predominantly affects older patients and is characterized by activation of DNA damage response and *TP53*-related pathways, together with enrichment of PD-1^+^ CD4^+^ T cells within the tumor microenvironment [50].

## 5. Role of EBV in HL Pathogenesis

The association between EBV and cHL varies considerably according to geographic region, age, and histological subtype. EBV positivity is reported in approximately 30–50% of cHL cases in the USA and Europe, whereas substantially higher rates, approaching 100%, have been observed in some developing countries, including Vietnam, Kenya, and Honduras [51]. EBV-positive cHL is more frequent in children (<10 years) and older adults (>60 years) and is strongly associated with the MC subtype. These epidemiological patterns may reflect differences in the timing of primary EBV infection, socioeconomic and environmental conditions, and age-related immune competence. Early EBV acquisition in developing countries may promote EBV-driven lymphomagenesis in children with relatively immature immunity, whereas immunosenescence and altered Th1/Th2 balance may contribute to the higher prevalence in older adults [2,51,52].

The interaction between EBV and the germinal center (GC) may represent an important step in EBV-associated lymphomagenesis. Roughan and Thorley-Lawson showed that EBV-infected B cells can reside within GCs while retaining GC-associated markers, including BCL6 and AID, supporting the GC as a potential pathogenic interface for EBV-associated lymphomas [53]. Moreover, Lacoste et al. demonstrated that ex vivo EBV infection of human B cells induces an early DNA-damage response and structural chromosomal abnormalities associated with telomere dysfunction, suggesting that EBV infection may contribute to genomic instability, although the relevance of these findings to HRS cells remains to be established [54].

EBV-infected HRS cells are typically characterized by the expression of EBV nuclear antigen 1 (EBNA1), latent membrane protein 1 (LMP1), and LMP2A. EBNA1 is essential for replication and maintenance of the viral genome [31]. LMP1 contains a cytoplasmic domain that functionally mimics constitutive CD40 signaling, thereby activating downstream pathways including NF-κB and PI3K/AKT/mTOR [55]. In addition, LMP1 can induce PD-L1 expression through activation of STAT transcription factors, providing an alternative mechanism of immune evasion [56].

Importantly, LMP1 may also contribute to the generation of the characteristic multinucleated RS-cell phenotype through disruption of telomere homeostasis. Knecht et al. showed that the transition from mononuclear Hodgkin cells to multinucleated RS cells is associated with progressive telomere dysfunction and formation of telomeric aggregates [57]. Mechanistically, Lajoie et al. demonstrated that LMP1 downregulates the shelterin proteins TRF1, TRF2, and POT1, promoting telomeric abnormalities and multinuclearity; restoration of TRF2 prevents LMP1-induced multinucleation, supporting a direct role of LMP1-mediated shelterin disruption in RS-cell formation [58].

Conversely, LMP2A harbors signaling motifs that mimic tonic BCR signaling and can sustain survival signals in GC B cells, although the downstream signaling cascades are not entirely identical to physiological BCR activation [59]. Further support for the oncogenic role of LMP1 comes from a humanized mouse model. Li et al. showed that infection with the EBNA2-deleted EBV strain P3HR1 can generate Hodgkin-like lymphomas with large, frequently multinucleated HRS-like cells and an LMP1-high type II latency program [60]. Although this model does not fully recapitulate human cHL, it provides in vivo evidence linking an LMP1-expressing EBV latency program to an HRS-like phenotype.

The pathogenetic role of EBV is further supported by the observation that EBV-positive cases less frequently harbor mutations in NF-κB pathway genes such as TNFAIP3 and NFKBIA, suggesting that viral oncogenic signaling may partially substitute for genetic alterations [17,40].

## 6. Tumor Microenvironment

A defining hallmark of cHL is its highly organized and functionally dynamic tumor microenvironment, which represents the major cellular component of the tumor tissue and is essential for HRS cell survival and expansion.

HRS cells are embedded within a heterogeneous infiltrate composed of CD4^+^ T helper and regulatory T cells, CD8^+^ T cells, natural killer cells, macrophages, eosinophils, mast cells, dendritic cells, fibroblasts, and endothelial elements, reflecting an intricate network of tumor–host interactions [40,61]. Among these populations, CD4^+^ T cells typically predominate and frequently form tight cellular aggregates around HRS cells, a phenomenon known as rosetting. This intimate contact is mediated by adhesion molecules such as CD54 on HRS cells and CD11a/CD18 on T cells, as well as by costimulatory receptor–ligand pairs including CD40–CD40L and CD80/CD86–CD28, which collectively provide essential survival and proliferative signals [62]. Additional cellular elements within the Hodgkin lymphoma microenvironment further sustain HRS cell growth and survival. For example, CD30 ligand expressed by mast cells and eosinophils can activate CD30 signaling in HRS cells. Reciprocally, HRS cells orchestrate their supportive niche through the secretion of cytokines and chemokines that enhance the recruitment, survival, and functional modulation of surrounding immune cells. Chemokines such as CCL5, CCL17 (TARC), CCL20, CCL22, and CCL28 promote the infiltration of T helper and regulatory T cells, macrophages, and eosinophils, whereas additional mediators contribute to stromal remodeling and angiogenesis [40,62].

Indeed, immune evasion represents a central functional dimension of the cHL microenvironment. Despite the presence of abundant immune infiltrates and a substantial neoantigen load, HRS cells frequently evade cytotoxic surveillance through multiple complementary mechanisms, including downregulation of MHC class I expression, expression of inhibitory ligands such as PD-L1, PD-L2, CD95L, HLA-E, and HLA-G, and secretion of immunosuppressive cytokines such as IL-10, TGF-β, macrophage migration inhibitory factor, and galectin-1. Furthermore, recruitment of FOXP3^+^ and LAG-3^+^ regulatory T cells and polarization of tumor-associated macrophages contribute to suppression of CD8^+^ T-cell and NK-cell function, often through PD-L1 expression and indoleamine-2,3-dioxygenase–mediated tryptophan depletion. Additional mechanisms, including purinergic signaling driven by CD38, CD39, and CD73, further reinforce the immunosuppressive milieu [40].

Beyond immune cell populations, recent studies have highlighted the importance of the stromal compartment of the cHL microenvironment, particularly cancer-associated fibroblasts (CAFs). Using multiplex immunofluorescence and spatial single-cell analysis, Leivonen and colleagues demonstrated that distinct CAF subsets exert divergent clinical effects. Whereas a higher overall CAF abundance was independently associated with favorable progression-free survival, enrichment of PDGFRB^+^ CAFs in close proximity to Hodgkin and Reed–Sternberg cells predicted inferior progression-free and overall survival, underscoring the importance of stromal heterogeneity and spatial organization in cHL [63].

## 7. Conclusions

In summary, HL represents a unique model of B-cell malignancy in which the biological identity of the tumor is shaped by the interplay between cell of origin, genetic lesions, and a complex tumor microenvironment. Although the malignant Hodgkin and Reed–Sternberg cells constitute only a minor fraction of the tumor mass, recurrent genetic alterations affecting key signaling pathways, including NF-κB, JAK/STAT, and PI3K/AKT, together with mechanisms of immune evasion drive lymphoma development and persistence. At the same time, extensive transcriptional reprogramming leads to the loss of the B-cell phenotype, highlighting the importance of epigenetic and regulatory mechanisms in defining the HRS cell identity. Recent advances in high-throughput sequencing technologies have substantially expanded our understanding of the genomic architecture of cHL and revealed biologically meaningful molecular subtypes.

These biological insights are increasingly informing therapeutic strategies. The efficacy of PD-1 blockade and CD30-targeted therapy highlights the clinical relevance of immune evasion and HRS-cell vulnerabilities, while emerging molecular insights may support the development of more targeted and personalized treatments.

Continued integration of genomic, immunological, and microenvironmental insights will be essential for refining biological classification, identifying novel therapeutic vulnerabilities, and ultimately enabling more precise and less toxic treatment strategies for patients with this highly curable yet biologically complex disease.

## Figures and Tables

**Table 1:** ctDNA-based molecular clusters in classical Hodgkin lymphoma.

Study	Sequencing	Clusters	Biology	Genomic Features	Prognostic Associations
Alig et al., Nature 2024 [20]	Targeted ctDNA seq + WES	2 (H1/H2)	Immune signaling vs. chromosomal instability	H1: high TMB; NF-κB/JAK-STAT/PI3K alterations. H2: high SCNAs, *TP53/KMT2D* alterations, high ctDNA, EBV enrichment.	H2 associated with inferior PFS
Heger et al., JCO 2024 [21]	Targeted ctDNA seq + WES	3 (Inflammatory immune escape, Virally-driven, Oncogene-driven)	Immune escape, viral biology, oncogenic signaling	inflammatory immune escape: 9p24.1/PD-L1 amplification. Virally driven: low TMB, EBV/HHV6. Oncogene-driven: high TMB; *TNFAIP3, ITPKB, SOCS1* mutations.	inflammatory immune escape HL had the poorest PFS; Oncogene-driven HL had the best PFS.
Pirosa et al., Blood 2025 [23]	Targeted ctDNA seq	2 (C1/C2)	Distinct genomic instability mechanisms	C1: high TMB, AID signatures, MSI-like features, frequent WGD. C2: chromosomal instability with extensive SCNAs.	No significant PFS difference between C1 and C2.

Abbreviations: ctDNA, circulating tumor DNA; WES, whole-exome sequencing; SCNA, somatic copy-number alteration; TMB, tumor mutational burden; EBV, Epstein-Barr virus; HHV6, human herpesvirus 6; AID, activation-induced cytidine deaminase; MSI, microsatellite instability; WGD, whole-genome duplication; PFS, progression free survival.

## Data Availability

No new data were created or analyzed in this study. Data sharing is not applicable to this article.
